# EXERCISE OR PHYSICAL ACTIVITY: WHICH IS MORE STRONGLY ASSOCIATED WITH THE PERCEPTION OF SLEEP QUALITY BY ADOLESCENTS?

**DOI:** 10.1590/1984-0462/;2018;36;3;00014

**Published:** 2018

**Authors:** Luciano Machado Ferreira Tenório de Oliveira, Alison Oliveira da Silva, Marcos André Moura dos Santos, Raphael Mendes Ritti-Dias, Paula Rejane Beserra Diniz

**Affiliations:** a Centro Universitário Tabosa de Almeida Asces-Unita, Caruaru, PE, Brasil.; b Universidade de Pernambuco, Recife, PE, Brasil.; c Universidade Nove de Julho, São Paulo, Brasil.; d Universidade Federal de Pernambuco, Recife, PE, Brasil.

**Keywords:** Physical activity, Exercise, Sleep, Adolescent, Atividade física, Exercício, Sono, Adolescente

## Abstract

**Objective:**

To analyze the association of exercises and physical activity with the perception of sleep quality by adolescents.

**Methods:**

This is a cross-sectional epidemiological survey with statewide coverage, whose sample was composed of 6,261 adolescents (14-19 years old) who were selected by random sampling of conglomerates. The Global School-Based Student Health Survey questionnaire was used for data collection. The chi-square test and the binary logistic regression were applied for data analyses.

**Results:**

In the sample, 29% of adolescents did not exercise and were not classified as physically active. Adolescents who did not exercise were more likely to present a negative perception of sleep quality (OR 1.13, 95%CI 1.04-1.28, p=0.043). No association between the level of physical activity and the perception of sleep quality was found (OR 1.01, 95%CI 0.89-1.14, p=0.868). Those who practiced exercises only had less chance of perceiving sleep quality as poor (OR 0.82, 95%CI 0.71-0.95). However, those who practiced exercise and had a physically active life had less chances of having a negative perception of their sleep (OR 0.79, 95%CI 0.68-0.93).

**Conclusions:**

Practicing physical activity alone was not enough to increase the chances of positive sleep quality perception. Only physical exercise had a positive association with sleep quality perception.

## RESUMO

**Objetivo:**

Analisar a associação do exercício físico e da atividade física com a percepção da qualidade do sono em adolescentes.

**Métodos:**

Trata-se de um estudo com abordagem quantitativa que integra o levantamento epidemiológico transversal de base escolar e abrangência estadual cuja amostra foi constituída por 6.261 adolescentes (14 a 19 anos), selecionados por meio de uma estratégia de amostragem aleatória de conglomerados. Os dados foram coletados a partir do questionário *Global School-based Student Health Survey*. O teste do qui-quadrado e a regressão logística binária foram utilizados nas análises dos dados.

**Resultados:**

Na amostra, 29% dos adolescentes não faziam exercício e não foram classificados como fisicamente ativos. Os adolescentes que não praticavam exercício físico tinham mais chances de apresentar uma percepção negativa da qualidade do sono (OR 1,13, IC95% 1,04-1,28; p=0,043). Não foi encontrada associação entre o nível de atividade física e a percepção da qualidade do sono (OR 1,01, IC95% 0,89-1,14; p=0,868). Ao serem avaliadas as práticas de forma isolada ou simultânea, constatou-se que aqueles que praticavam exercício físico apresentavam menor chance de terem uma percepção negativa da qualidade do sono (OR 0,82, IC95% 0,71-0,95) e, ao praticarem exercício e, paralelamente, terem uma vida fisicamente ativa, essas chances diminuíam ainda mais (OR 0,79, IC95% 0,68-0,93).

**Conclusões:**

Ser classificado como fisicamente ativo, por si só, não foi suficiente para uma melhor percepção da qualidade do sono, pois apenas a prática de exercício físico apresentou tal associação.

## INTRODUCTION

Adolescence is marked by changes in sleep pattern that seem to reflect both in amount and quality.^1^ ^,^ ^2^ ^,^ ^3^ ^,^ ^4^ ^,^ ^5^ In this context, studies have reported an association between sleep quality and some indicators of physical and mental health, ^1^ ^,^ ^2^ ^,^ ^3^ becoming an important feature for the health and well-being of adolescents.^1^ ^,^ ^2^ ^,^ ^4^

Changes in sleep pattern and quality during adolescence are attributed to the physiological, environmental, social and behavioral changes in this phase of life.^3^ ^,^ ^4^ ^,^ ^6^ When it comes to behavioral factors specifically, practicing exercises has been pointed out as a factor associated with better sleep quality in adolescents.^7^ Brand et al.^8^ studied a sample of adolescents and reported that regular practice of exercises is related to better sleep quality in 38 subjects aged 18 years. Another study showed a single exercise session accounting for approximately 50% of improved sleep quality in adolescents.^7^

Over the past few years, studies have been focusing on identifying the role played by daily physical activities - commuting, occupational activities, leisure, etc. - and regular practice of exercises on the sleep quality of adolescents.^9^ ^,^ ^12^ Important to emphasize that physical activity and exercises are distinct practices and should be evaluated separately: the former is related to any movement resulting in energy expenditure above resting levels, while the latter are planned, structured and repetitive activities aiming at the maintenance or optimization of physical conditioning.^13^ However, findings seem somewhat uncertain and controversial due to other behavioral and social factors that may function as mediators in this complex association.^11^

Therefore, identifying this interaction between physical activity or exercises and sleep quality of adolescents is a gap that needs better filling in. The objective of this study was to analyze the association of exercises and physical activity with the perception of sleep quality in adolescents.

## METHOD

This is a quantitative study that integrates the school-based cross-sectional epidemiological survey with statewide coverage called “Practice of physical activities and health-risk behaviors in high-school students in the State of Pernambuco”^( )^. The research protocol was approved by the Research Ethics Committee of Universidade de Pernambuco (UPE) (CAAE 0158.0.097.000-10/CEP-UPE: 159/10). All the determinations of Resolution nº 196/96 by the National Health Council (CNS) were complied with. In addition, the present study was approved by the State Secretariat of Education and Culture (SEDUC) of the State of Pernambuco, which also provided data for the school census.

The target population, estimated at 373,386 subjects according to SEDUC data, was made up of high school students aged 14 to 19 years and enrolled in the state public school network. The following parameters were adopted to calculate sample size: 95% confidence interval (95%CI); maximum tolerable error of 2%; sampling design effect of two; and prevalence estimated at 50% (adopted based on the multiple factors analyzed in the study). From these parameters, the sample size was estimated in 5,683 students. Students were selected by two-stage cluster sampling.

In the first stage, the primary sample units were randomly selected schools, considering the distribution proportionality in 17 microregions of the State. Regional distribution was considered as the number of schools located in the area covered by each of the 17 Regional Education Offices (GRE). Schools were sorted according to the number of students enrolled in high school based on the following criteria: small (<200 students); medium (200 499 students); and large (>500 students). Students enrolled in the morning and afternoon periods were grouped into a single category (daytime students). All students in the randomized groups were invited to participate in the study.

Data were collected between the first (May and June) and the second semester (August, September, October and November) of 2011, using the Global School-Based Student Health Survey (GSHS), by the Organization World Health Organization (WHO)^14^ - previously validated and commonly used in research with adolescents.^15^ ^,^ ^16^ Prior to data collection, a pilot study was conducted in order to test the applicability of the instrument. Data were collected at a reference school of the state public school network in the city of Recife, Pernambuco, with a sample of 86 adolescents aged 14 to 19 years. Indicators of reproducibility pointed to a moderate to high intraclass correlation coefficient in most items of the questionnaire, with coefficients of agreement (kappa index) ranging from 0.52 to 1.00.

Questionnaires were applied to students in their classrooms in the form of a press conference without the presence of teachers. Students were advised by two previously trained applicators, who clarified queries and assisted them in completing the data. All students were informed that their participation was voluntary and that the questionnaires were not to contain any type of personal identification. Students were also informed that they could drop out at any stage of data collection. An informed consent form was used to obtain permission from parents of students aged below 18 years old so they could take part in the study. Those who were 18 years old and up signed the form and gave their agreement to participate in the study.

The dependent variable of the study (sleep quality perception) was measured by the following question: “How do you evaluate the quality of your sleep?”. Answers were dichotomized in “positive” for those who rated their sleep quality as “good”, “very good” or “excellent”, and “negative” for those who rated it as “poor” or “fair”. Hours of sleep was measured from the following question: “How many hours do you sleep on average during the night?”, with answer options in hours.

Regarding the level of physical activity, two questions in GSHS were considered:

1. “During the past 7 days, how many days have you been physically active for at least 60 minutes?”
2. “For 1 typical or normal week, on how many days are you physically active for at least 60 minutes?”

In order to estimate the level of physical activity, the procedure suggested by Prochaska, Sallis and Longo^17^ was adopted for questions 1 and 2 using the following formula: (Question 1 + Question 2) ÷ 2. If the result was less than five days, the adolescents were considered insufficiently active, that is, not matching recommendations of physical activity. It should be noted that this questionnaire was validated and considered acceptable when compared with accelerometry, in addition to being commonly used in research with adolescents.^18^ ^,^ ^19^ Practice of physical exercises was determined by the question: “Do you regularly perform any type of activity in your free time, such as exercise, sports, dance or martial arts?”, with dichotomous answers (“yes” or “no”).

Personal information, socioeconomic and sociodemographic variables were obtained by direct questions related to gender, age, skin color, marital status, place of residence, occupation and mothers’ schooling: “What is your sex?”; “How old are you, in years?”; “Do you consider yourself white, black, brown, yellow or indigenous?”; “What is your marital status?”; “Your residence is located in the urban or rural area?”; “Do you work?”; and “Check the alternative that best indicates the educational level of your mother”, respectively.

Data were tabulated in EpiData (version 3.1). The “Check” function was used to electronically control data input in typing phase. In order to detect and correct errors, the data entry was repeated, the files were compared and errors were corrected.

Data analysis was made in Statistical Package for the Social Sciences^®^ (SPSS) for Windows, version 16.0 (IBM Corp., Armonk, New York, USA). In the descriptive analysis, frequency distribution was considered. In inferential analysis, the Pearson’s chi-square test was used to evaluate the isolated association between the perception of sleep quality and practice of exercise and physical activity. A binary logistic regression was made by estimating Odds Ratio (OR) and 95% confidence intervals (95%CI) to express the level of association between independent variables (exercise and physical activity) and the dependent variable (perception of sleep quality), adjusting for possible confounding factors (nutritional status, gender, age and economic condition). After predictor variables of the final model were obtained, occurrence of interaction between genders was tested. For confounding variables, only those with statistical significance lower than 0.20 in the univariate analysis (p<0.20) were added to the model, being simultaneously introduced. Thus, the Backward method led us to the final regression model with only variables presenting significant contribution to the model.

## RESULTS

Among 7,528 students who were in classroom on the day of data collection, 317 refused to participate and 16 were not authorized by parents or guardians to participate, totaling 333 (4%) refusals. Therefore, 7,195 students were interviewed and assessed. After exclusion of 15 questionnaires not properly filled in and 919 (12.8%) whose subjects were outside the stipulated age range, the final sample had 6,261 adolescents aged 14 to 19 years (16.6 ± 1.3 years), females being 59.7% of them. The socioeconomic and demographic characteristics, as well as prevalence related to the perception of sleep quality, practice of exercise and physical activity by adolescents are presented in Table 1.

**Table 1:** Socioeconomic, demographic data and prevalence of sleep quality perception, exercise and physical activity among high-school students enrolled in the Pernambuco State public school network.

	Total	
	(n=6,261)	
	n	%
Gender		
Male	2,524	59.7
Female	3,737	40.3
Age (years)		
14-15	1,350	21.6
16-17	3,344	53.4
18-19	1,567	25.0
Occupation		
Works	1,388	22.2
Does not work.	4,856	77.8
Skin color		
White	1,620	26.0
Other	4,619	74.0
Living area		
Urban	4,644	74.5
Rural	1,587	25.5
Mother’s educational level		
More than 8 years of study	1,903	35.3
Less than or 8 years of study	3,488	64.7
Perception of sleep quality		
Positive	4,694	75.0
Negative	1,561	25.0
Hours of sleep		
More than 8	3,573	57.2
Less or 8	2,672	42.8
Nutritional status		
Eutrophic	5,072	83.5
Overweight	742	12.2
Obese	262	4.3
Physical activity level		
Active	2,192	35.1
Insufficiently active	4,047	64.9
Exercises		
Practices	4,007	64.0
Does not practice	2,251	36.0

**Figure 1:**
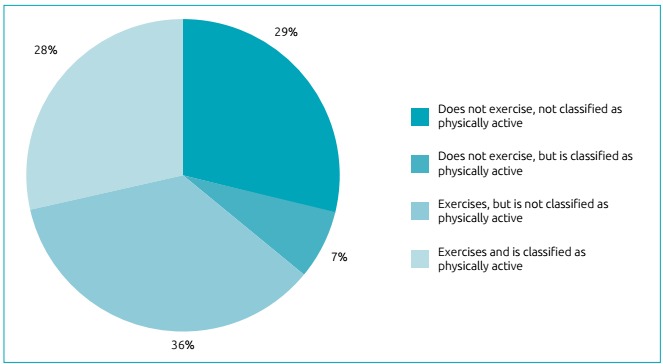
Prevalence of practice of exercises as to levels of physical activity among high-school students enrolled in the State public network of Pernambuco.

Figure 1 presents values classifying the level of physical activity and practice of physical exercises by adolescents in an isolated or simultaneous way; 29% of adolescents did not exercise and were not classified as physically active, and 28% exercised and were classified as physically active.

After adjustment, those who practiced physical exercise presented lower chances of perceiving their sleep quality negatively (OR 0.82, 95%CI 0.71-0.95), and, when they reported exercising and having a physically active life, chances were even lower (OR 0.79, 95%CI 0.68-0.93), when compared to those who did not exercise and were not classified as physically active. Being classified as physically active only was not sufficient to reduce chances of negative perception of sleep quality (p=0.841) (Table 2).

**Table 2:** Odds Ratio for physical activity versus exercises as related to negative perception of sleep quality among high-school students enrolled in the Pernambuco State public school network.

Exercise	Poor perception of sleep quality			
*versus*	*Odds Ratio*	95%CI	p-value	p-value
Physical activity level	(adjusted)^a^			overall
Does not exercise and is not classified as physically active.	1			0.001*
Does not exercise but is classified as physically active.	0.98	0.77-1.24	0.841	
Exercises, but is not classified as physically active.	0.82	0.71-0.95	0.010*	
Exercises and is classified as physically active.	0.79	0.68-0.93	0.004*	

95%CI: 95% confidence interval; ^a^Adjusted for age, gender, nutritional status and hours of sleep; *p<0.05.

## DISCUSSION

The objective of this study was to analyze the association of exercise and physical activity with the perception of sleep quality by adolescents. The main results show that a quarter of adolescents reported having a negative perception of their sleep quality. The practice of physical exercises alone, not physical activity, was associated with better perception of sleep quality, but added to a physically active life, it decreases the chances of perception of poor quality, regardless of age, gender, nutritional status, and number of hours of sleep.

Our findings corroborate those reported in a study conducted with adolescents from 20 public schools in Recife and Florianópolis, whose transversal and longitudinal data accounted for 45.7% and 45.8% of adolescents reporting a negative perception of their sleep quality, respectively.^6^ Another study carried out in the state of Santa Catarina between 2001 and 2011 showed an increase in prevalence of negative perception of sleep quality in 31.2% of adolescents.^20^ The authors suggest that this can be explained by the changes in sociodemographic factors, which, in turn, were influenced by the technological evolution over the decade in which the study was conducted. The high percentage found for poor sleep quality in Brazilian adolescents reflects the importance and necessity of public health organizations creating strategies and actions that promote a healthier lifestyle.

Interestingly, in this study that, even when practicing physical exercises, the individual can be classified as insufficiently active, and those who are classified as physically active do not have to necessarily exercise. One can state that 7% of adolescents did not practice exercises, but were classified as physically active, and 36% did practice exercises, but were not classified as physically active. Important to note that physical activity can be understood as an inherent human behavior, for any and all voluntary body movement produced by the skeletal muscles that result in energy expenditure above resting levels is considered physical activity.^13^ In order for one to be classified as physically active, they should perform 60 minutes or more of moderate to vigorous physical activity daily^5^ or perform it on 5 or more days within the week.^21^ Exercises are related to intention of movement, considering that they are a subgroup of planned, structured and repetitive physical activities ,aiming at the maintenance or optimization of physical conditioning.^13^ Conceptually and operationally, physical activity and exercises are distinct, which reinforces the importance of separately evaluating their influence on sleep quality.

In the present study, the practice of physical exercise, not physical activity, was associated with a better perception of sleep quality, regardless of age and sex of adolescents. Studies on physical activity and sleep quality among adolescents have found controversial results, indicating that behavioral and sociodemographic factors can mediate this relationship.^11^ On the other hand, being systematically involved in physical exercise programs that promote greater energy expenditure, with controlled intensity, frequency and duration present results that somewhat seem to be more robust.^22^

In a recent meta-analysis, ^22^ being involved in physical exercises was shown to be beneficial to sleep quality and to decrease both sleep latency and sleeping medication. In another study, Noland et al.^23^ reported the practice of pre-bedtime physical exercise being cited as one of the strategies used by adolescents to fall asleep. On the other hand, according to Santiago et al.,^7^ performing a strength training session in the morning or the afternoon improved the sleep quality of adolescents in a boarding school.

Theoretical models that seek to explain the effects of exercise on sleep are associated with thermoregulatory hypotheses, energy conservation and body restoration. The thermoregulatory hypothesis states that the increase in body temperature resulting from physical exercises would favor sleep triggering through the stimulation of body heat dissipation mechanisms controlled by the hypothalamus, and the increase in slow-wave sleep, the deep sleep phase in which physical restoration takes place.^24^ ^,^ ^25^ The hypothesis of energy conservation consists in the increase of energy expenditure resulting from the practice of physical exercises during wakefulness. This increase leads the individual to require sleep as a means to remedy energetic balance for the coming wake hours.^24^ ^,^ ^25^ The restorative hypothesis assumes that the increase in catabolism resulting from exercises entails a decrease in energy reserves and results in an increased need for sleep to achieve anabolism.^24^ ^,^ ^25^

It was interesting to note in this study that adolescents who exercised and had a more physically active life had a greater chance of perceiving their sleep positively. Even in face of the results found, caution is required in extrapolating the findings, since the study presents some limitations that should be considered. The cross-sectional design and the correlative nature of data prevent us from establishing a causal relationship between practice of exercises and perception of sleep quality. In addition, sleep quality, physical activity level and physical exercise levels were obtained by indirect measurement, the authors being aware that direct methods (actigraphic, polysomnographic, and accelerometry tests) would provide more accurate information. Further studies should, therefore, use direct methods in addition to longitudinal interventions related to exercise and control the intensities applied, as this variable can directly influence sleep. Even knowing the limitations related to the use of the questionnaire, a sample with more than 6,000 individuals would make the use of direct evaluations unfeasible due to convenience and cost.

The strengths, we could mention sample size, the sampling procedures, which were established to ensure its composition by adolescent attending schools in rural and urban areas and in different shifts. The analysis controlled for potential confounding factors should also be stressed out.

In conclusion, being classified as physically active was not enough for a better perception of sleep quality, since only the practice of physical exercise was associated with the best perceptions of sleep quality, regardless of gender, age, nutritional status and number of hours of sleep among adolescents.
